# Gas6/TAM Signaling Components as Novel Biomarkers of Liver Fibrosis

**DOI:** 10.1155/2019/2304931

**Published:** 2019-09-08

**Authors:** Carlo Smirne, Cristina Rigamonti, Carla De Benedittis, Pier Paolo Sainaghi, Mattia Bellan, Michela Emma Burlone, Luigi Mario Castello, Gian Carlo Avanzi

**Affiliations:** ^1^Internal Medicine Division, Maggiore della Carità Hospital, Department of Translational Medicine, Università del Piemonte Orientale (UPO), Novara, Italy; ^2^Immunorheumatology Unit, CAAD (Center for Autoimmune and Allergic Diseases), “Maggiore della Carità” Hospital, Novara, Italy; ^3^IRCAD (Interdisciplinary Research Center of Autoimmune Diseases), Novara, Italy

## Abstract

Liver fibrosis consists in the accumulation of extracellular matrix components mainly derived from activated hepatic stellate cells. This is commonly the result of chronic liver injury repair and represents an important health concern. As liver biopsy is burdened with many drawbacks, not surprisingly there is great interest to find new reliable noninvasive methods. Among the many are new potential fibrosis biomarkers under study, some of the most promising represented by the growth arrest-specific gene 6 (Gas6) serum protein and its family of tyrosine kinase receptors, namely, Tyro3, Axl, and MERTK (TAM). Gas6/TAM system (mainly, Axl and MERTK) has in fact recently emerged as an important player in the progression of liver fibrosis. This review is aimed at giving an overall perspective of the roles played by these molecules in major chronic liver diseases. The most promising findings up to date acknowledge that both Gas6 and its receptor serum levels (such as sAxl and, probably, sMERTK) have been shown to potentially allow for easy and accurate measurement of hepatic fibrosis progression, also providing indicative parameters of hepatic dysfunction. Although most of the current scientific evidence is still preliminary and there are no in vivo validation studies on large patient series, it still looks very promising to imagine a possible future prognostic role for these biomarkers in the multidimensional assessment of a liver patient. One may also speculate on a potential role for this system targeting (e.g., with small molecule inhibitors against Axl) as a therapeutic strategy for liver fibrosis management, always bearing in mind that any such therapeutic approach might face toxicity.

## 1. Introduction

### 1.1. Hepatic Fibrosis: Pathophysiology and Clinical Importance

All hepatologists wish they had a crystal ball in their clinic to enable them to determine whether or not their immediate patient has liver fibrosis or not. This is because liver fibrosis is a predominate key component of essentially all chronic liver diseases. It is the formation of scar tissue in response to parenchymal injuries such as chronic hepatitis B (CHB) and C (CHC), nonalcoholic fatty liver disease (NAFLD), or alcoholism (ALD). The continuous and progressive replacement of hepatocytes by the extracellular matrix and fibrous tissue eventually leads to liver cirrhosis, which in turn may lead to liver failure or promote a conducive microenvironment for cancer development, in particular hepatocellular carcinoma (HCC) [1, 2]. Whatever the etiology of liver injury, it is the activation of hepatic stellate cells (HSCs) that is responsible for liver fibrosis, being HSCs the main collagen-producing cells in the damaged liver [3, 4]. HSCs transform during chronic liver injuries from a quiescent state into a myofibroblast-like phenotype (HSCs/MFBs), which proliferate and migrate towards areas of necrosis and regeneration [5, 6]. The main action of HSCs/MFBs consists in a profound alteration of the extracellular matrix (ECM) composition due to the upregulation of proteins such as *α*-smooth muscle actin (*α*-SMA), interstitial collagens such as collagen 1A1, and matrix metalloproteinases (MMPs) such as MMP9 as well as tissue inhibitors of metalloproteinases (TIMPs), and proteoglycans. Activated HSCs also generate hepatic cytokines such as transforming growth factor-*β*, platelet-derived growth factor, connective tissue growth factor, fibroblast growth factor, hepatocyte growth factor, and vascular endothelial growth factor and recruit inflammatory mono- and polymorphonuclear leukocytes that produce chemokines, including monocyte chemotactic protein- (MCP-) 1, regulated on activation normal T cell expressed and secreted (RANTES), chemokine (C-C motif) ligand 21 (CCL21), and C-C chemokine receptor type 5 (CCR5). Although HSCs' critical role in liver fibrosis was proposed nearly two decades ago [7], more recent data demonstrate that, regardless of the underlying etiology of liver disease, the majority of myofibroblasts comes from the liver-resident HSC population [8]. While liver fibrosis was once broadly thought of as an irreversible process, there is now substantial evidence that, at least from a speculative point of view, a near-normal hepatic architecture can be restored upon cessation of injury [9]. However, these promising findings must be offset by the fact that, after cessation of the fibrotic triggering insult, around half of the activated HSCs survive in an apparently quiescent state, as they are primed to quickly reactivate into myofibroblasts in response to fibrogenic stimuli [10, 11]. This leaves room for doubt that antifibrotic therapies meant to inhibit activated HSCs, although beneficial to prevent ECM deposition, may be sufficient to revert fibrosis permanently.

In any case, accurately defining the current fibrosis stage reached by a patient along the course of his/her disease is, as previously mentioned, of quintessential clinical importance, since crucial decisions, such as starting monitoring for complications (e.g., esophageal varices or HCC), depend on it. Moreover, the presence and extent of liver fibrosis help to predict prognosis and to prompt treatment decisions in various chronic liver diseases. For instance, different international treatment guidelines mention that the severity of liver fibrosis should be considered, regardless of serum alanine aminotransferase level, for starting antiviral treatment for CHB [12, 13]. In conclusion, there are a multiplicity of reasons for which it is crucial to diagnose and assess the extent of liver fibrosis.

### 1.2. Liver Biopsy for Staging of Fibrosis

Liver biopsy is still considered as the gold standard method to assess liver fibrosis; moreover, it provides useful information about diagnosis as well as other damaging processes such as necrosis, inflammation, and steatosis [14]. All most widely used methods to assess histological fibrosis are based on the description of the elements that mark the progression of the disease, such as periportal fibrosis, septal fibrosis, and/or nodule formation [15].

One obvious and insurmountable limitation of liver biopsy is that it is perceived as unduly invasive.

Furthermore, an insufficient sample size and divergence based on differing experience among pathologists can lead to significant interobserver disagreement. Risks associated with liver biopsy include pain (84%), bleeding (0.5%), damage to the biliary system (0.2%), and infections (0.1%), with a mortality rate of approximately 0.01% [16]. Finally, the cost of liver biopsy can be significant, leading to a questionable cost-effectiveness ratio [17].

### 1.3. Current Clinical Noninvasive Techniques to Assess Hepatic Fibrosis

These limitations of liver biopsy have given urgency for the development of alternative diagnostic procedures for liver fibrosis. As a result, noninvasive techniques have gained popularity in current clinical settings, leading to a reduction of liver biopsies to stage the degree of liver fibrosis; however, they also have several pitfalls.

The most traditional alternatives to invasive procedures are represented by medical imaging. Ultrasonography, for example, can suggest the presence of fibrosis and cirrhosis but it is neither sensitive nor specific in its implementation, performing positively only in late stages of liver cirrhosis, when the signs of portal hypertension develop [18]. Computed tomography and magnetic resonance are more sensitive and specific but are burdened by the association of high costs and inadequate interrater reliability among different radiologists; moreover, the extensive use of computed tomography scan is limited by radiological risks [19].

As a result, more innovative noninvasive approaches have been (and are being) designed. The two principal approaches that have been validated in large patient cohorts with various etiologies are elastographic techniques measuring liver stiffness and the detection and quantification of serum markers.

Elastographic methods, which test liver stiffness, are represented mainly by transient elastography (FibroScan®). Alternative techniques include point or multidimensional shear wave elastography and magnetic resonance elastography. All of these procedures, in addition to requiring expensive equipment, may be inaccurate in obese or ascitic patients and may lead to overestimation of fibrosis in patients with high necroinflammatory activity [20]. Moreover, being the result of the sum of inflammation and fibrosis in the liver parenchyma, liver stiffness *per se* may not be the ideal candidate to monitor for fibrosis regression.

The advantages of biomarkers over liver biopsy, besides being minimally invasive, are, at least from a speculative point of view, their cost, ease of application, interlaboratory reproducibility, and broad availability [21]. The rationale of their use derives from the notorious ability of the liver to either produce or modify a multiplicity of chemicals, a property that has long been explored to estimate, from the changes in their blood concentration, the degree to which liver function is impaired and/or to which extent organ damage is in addition to monitoring therapies. Indeed, liver biochemistry panels (e.g., aminotransferases, alkaline phosphatase, *γ*-glutamyl transferase, bilirubin, albumin, and prothrombin time) are included in almost all laboratory routines, being informative, relatively cheap, and prone to repeat testing [22]. Conceptually, fibrosis is of no exception. By-products spilling in the blood as a result of the deposition and removal of ECM produced by HSCs and other hepatic cells can be taken as a proxy measure of what is occurring in the liver parenchyma and are generally referred to as direct markers of fibrosis (as opposed to the aforementioned markers for liver injury which are considered indirect markers of the same). Typically, serum levels of the former markers are elevated with progressing fibrosis and have a tendency to decrease with response to treatment [23]. As a result, their assessment may be useful for bringing about effective treatment, but they are neither organ specific nor readily available, unlike what would be required for an ideal biomarker [21]. An exhaustive classification according to their molecular structure can be found in a recent review from Nallagangula et al. [24].

As a general rule, although a single direct marker may serve as an indicator of disease severity, there is growing consensus that a combination of multiple markers as an integrated panel will enhance the performance characteristics in terms of specificity and sensitivity. This is why patients can now be profiled based on artificial intelligence algorithms that produce scores by combining different biochemical parameters (e.g., direct and/or indirect fibrosis markers and/or blood platelet count), including, in some cases, demographics such as age or gender. Some of the main scoring systems for liver fibrosis which have been implemented in clinical practice include the aspartate aminotransferase-to-platelet ratio (APRI) [25, 26], fibrosis- (FIB-) 4 [27], Fibro index [28], Bonacini index [29], Forns test [30], and NAFLD fibrosis score [31]. Unfortunately, none of these approaches have produced highly accurate results for liver fibrosis assessment to date [32] and their use in clinical practice is not comparable to those of prognostic scores, such as the Child-Pugh-Turcotte classification system [33] and the Model for End-stage Liver Disease score [34]. In this context, we also need to account for some derived scores, such as FibroTest [35], Fibrometer [36], Hepascore [37], and enhanced liver fibrosis [38], which include more specific blood tests such as direct fibrosis markers (e.g., hyaluronic acid [36–38], procollagen III amino terminal peptide [38], and tissue inhibitor of metalloproteinase 1 [38]), that are not routinely available. Again, the lines of evidence about their reliability and cost-effectiveness are not sufficient to support their use in clinical practice.

### 1.4. Evolving Biomarker Candidates for Liver Fibrosis

The aforementioned limitations of most current surrogate markers of liver fibrosis to provide stepwise follow-up (meaning a sensitive and specific manner for the detection and differentiation between the various stages of liver fibrosis and the possibility to detect modest progression or regression of fibrosis) explain why, on the one hand, liver biopsy has not yet been abandoned and why, on the other, there is great cultural eagerness to find new reliable noninvasive indicators, also due to the putative treatments for liver fibrosis appearing on the horizon. All newly discovered candidate markers may therefore play a vital role in the assessment of chronic liver injury which needs further evaluation. However, statistical comparison should always be made with established biomarkers and panels in large-scale multietiology validation studies [24, 39].

Among the many new potential markers which are under study, one of the most promising is represented by growth arrest-specific gene 6 (Gas6) serum protein and its family of receptors, namely, Tyro3, Axl, and MERTK (TAM). This system has long been demonstrated to have a pivotal role in fibrogenesis and in the progression of chronic liver diseases, yet it is believed that we are currently verging on a breakthrough in research due to the increasing knowledge of the fine interplay of these factors with the various mechanisms involved in liver damage. There is in fact a growing consent on its potential use also as a useful novel biomarker for the detection of liver fibrosis in vivo. However, many controversies remain due to the complexity of the biological systems involved.

The purpose of this work is to precisely review the literature data, highlighting the areas where the current lines of evidence are more concrete and those that still need further confirmation or validation.

## 2. Gas6/TAM Receptors

### 2.1. Biology of Gas6/TAM Receptors

TAM is one of the twenty subfamilies of receptor tyrosine kinases [40]. Members of the TAM receptor family are Tyro3, Axl, and myeloid-epithelial-reproductive tyrosine kinase (MERTK). All comprise two immunoglobulin-like and two fibronectin type III repeats in their extracellular domains in tandem. These are connected to a single-pass transmembrane domain and a cytoplasmic protein tyrosine kinase. Upon ligand binding, the receptor dimerizes and the tyrosine kinase becomes activated [41, 42]. TAM receptors differ in the physiological tissue expression. Axl is expressed in a wide variety of tissues and organs including the hippocampus, cerebellum, heart, skeletal muscle, liver, kidney, testis, brain, monocytes, macrophages, platelets, endothelial cells, and dendritic cell.

MERTK expression is found in the ovary, prostate, testis, lung, retina, and kidney and macrophages, dendritic cells, natural killer cells, megakaryocytes, and platelets. Tyro3 is most prominent in the nervous system, but it is expressed also in the ovaries, testis, breast, lung, kidney, osteoclasts, retina, monocytes/macrophages, and platelets [43]. Noteworthily, each receptor can be produced also as a soluble form (sAxl/sMERTK/sTyro3) [44].

First cloned in 1991, TAM receptors were all considered orphan receptors until 1995 [45]. In that year, their vitamin K-dependent ligands, protein S and Gas6, were identified [46–48]. While both Gas6 and protein S share common features of domain organization and both require dimerization and *γ*-carboxylation for their activity as TAM ligands, they have differential specificities and affinities to TAM receptors following their markedly different affinities. It is now generally accepted that Gas6 activates Axl, Tyro3, and MERTK and that protein S activates MERTK and Tyro3. More in detail, Axl is preferentially activated by Gas6 with 100–1,000x higher binding affinity over Tyro3 and MERTK, suggesting that it may be the most relevant of the three receptors for Gas6 in many tissues, whereas affinity between protein S and Axl has never been shown. MERTK displays lower sensitivity to both ligands, and it is observed to have the greatest phosphatidylserine (PS) dependence on ligand-induced activation. Tyro3 is preferentially activated by protein C. Moreover, Tyro3 and MERTK biological activation is enhanced in the presence of PS, implicating mainly both these receptors in the clearance of apoptotic cells.

Whatever the receptor, in many cells, the activation of TAMs is coupled with the downstream activation of the phosphoinositide 3 kinase (PI3K)/AKT pathway. Most of this downstream PI3K signaling is nucleated through a TAM-autophosphorylated Grb2-binding site, which is located 18 residues carboxy terminal to the kinase domain and is conserved in all three TAMs. Coupling to phospholipase C, ERK1/2, Ras, and MAP kinase activation have also been described in many different cells [43, 49–51].

Coming back to the two TAM ligands, it is important to note that they differ in tissue expression patterns. More in detail, while natural anticoagulant protein S is mainly synthesized in the liver, Gas6 is produced predominantly in the heart, kidneys, and lungs and, to a lesser extent, in the liver. Other important tissues where Gas6 is expressed are endothelial cells [52], vascular smooth muscle cells [53], and bone marrow [54]. Gas6 has also been shown to be present in murine platelets, but this presence in humans has been debated [55]. From a morphological point of view, the two proteins share a high structural homology and sequence identity. However, they have clearly different biological roles [56, 57]. Protein S has mainly a TAM-independent inhibitory effect on hemostasis [58–60]. The Gas6/TAM system has instead clearly emerged from basic and clinical studies to have rather pleiotropic effects with many biological functions, sometimes playing more than one role at a time, as frequently seen in human biology [61]. Specifically regarding the area of coagulation, Gas6 seems to stimulate hemostasis playing a complementary role in platelet function [62], and it has been proposed as a biomarker for the diagnosis of pulmonary embolism [63]. But, in recent years, several other signaling functions of TAM receptors have been described, such as stimulation of cell growth and proliferation, inhibition of apoptosis [53, 64], mediation of efferocytosis (e.g., the process by which dying cells are removed by phagocytes) [65], and modulation of inflammation [66]. These effects probably explain why the overexpression of TAMs (mostly Axl and MERTK) can drive conventional oncogenic signaling and survival pathways in different types of cancers, while also playing an important role in epithelial to mesenchymal transition and metastasis [42]. As a consequence, the overexpression of TAM receptors has been associated with chemoresistance and poor survival outcomes [67].

A current research field that deserves a separate discussion is the activity of Gas6/TAM on the immune system [68]. Gas6 activation of the TAM receptors (specifically, MERTK and Axl isolated from circulating monocytes and tissue macrophages) was initially found to inhibit Toll-like receptor (TLR) signaling, which in turn is a known trigger of rapid inflammatory cytokine production in various cell types [69]. Conversely, TLR signaling was demonstrated to markedly decrease Gas6 expression in mouse macrophages through the activation of the nuclear factor-*κ*B, further facilitating—in a self-regulatory mechanism—the TLR-mediated inflammation [70]. Furthermore, the Gas6/TAM system was shown to be directly involved in the clearance of apoptotic bodies [71]. As a matter of fact, Gas6 recognizes phosphatidylserine, a lipid normally expressed on the inner face of the plasma membrane and exposed on the external membrane during apoptosis, and bridges it with the TAM receptors, driving macrophages to the recognition of apoptotic cells and to their subsequent phagocytosis, stimulating natural killer cell development [72]. Other recent studies revealed that Gas6/TAM signaling is involved in inflammation by enhancing interactions between endothelial cells and leukocytes [73]. Moreover, the induction of Axl limits cytokine synthesis in activated monocytes or dendritic cells [74]. Based on these premises, it is not surprising that there has been speculation on its possible role for the system in preventing autoimmunity [75]. On the contrary, defects in TAM signaling have been associated with numerous autoimmune diseases and degenerative diseases, since an impaired clearance of apoptotic bodies and an inappropriate inflammatory response are considered critical for the deranged immune response observed in these conditions. The role of TAM receptors has been for instance studied in diseases such as rheumatoid arthritis [76], multiple sclerosis [77–79], systemic lupus erythematosus [80], Sjögren syndrome [81], and Alzheimer's disease [82].

The complexity of the crosstalk between Gas6 and its receptors has increased to a further extent by the fact that in many of the aforementioned diseases, such as rheumatoid arthritis and lupus erythematosus, an impairment of the physiological balance between the transmembrane and the inactive soluble form of the receptors has been observed, suggesting that an increased cleavage of the receptors could have biological relevance in the pathogenesis of these conditions [76, 83]. The most studied is probably sAxl. Physiologically, Axl is cleaved by a disintegrin and metalloproteinase (ADAM) 10 and 17 in a protein kinase C-dependent fashion causing the release of sAxl which maintains the ability to interact with Gas6 [84, 85]. Thus, the release of sAxl and its involvement in a negative feedback loop by Gas6 binding together with the *γ*-secretase-mediated release of a sAxl intracellular domain (ICD) suggest bidirectional signaling.

### 2.2. Role of Gas6/TAM under Healthy and Pathological Conditions in the Liver

In recent years, the Gas6/TAM interaction has been described to be relevant in inflammatory and healing processes of the liver; in fact, Gas6 globally seems to play a protective role in response to liver injury.

In the liver, Gas6 is mainly expressed in Kupffer cells with levels below those observed in other tissues such as those found in the lung, kidney, or heart [56]. However, after specific liver injury, other hepatic cell types may participate in its production. For instance, Gas6 produced by HSCs together with its receptor Axl participate in the signaling involved in the injury repair mechanisms. Moreover, it has been shown in animal models that Gas6 expression is also significantly upregulated in injured areas by the other key cellular actors involved after acute or chronic liver damage, such as macrophages, HSCs/MFBs, and liver progenitor cells (LPCs). In this context, Gas6 exerts an antiapoptotic effect on both HSCs and HSCs/MFBs, acting as a survival factor, probably supporting transient HSC/MFB accumulation during liver healing [86]. For instance, Gas6 produced by HSCs and infiltrating macrophages together with its receptor Axl participate in the signaling (which includes, among others, the aforementioned Axl/PI3K/AKT pathway) involved in the wound healing response to liver injury by carbon tetrachloride (CCl_4_), and LPCs induce Gas6 production after hepatectomy [86–88]. Moreover, an early increase in serum Gas6 levels has been demonstrated following liver ischemia/reperfusion (I/R) exposure [89].

Consistent with these findings, in Gas6−/− knockout (KO) mice, abnormal wound healing after CCl_4_-induced liver damage compared with wild-type animals has been reported, with decreased expression of activation markers for Kupffer cells (such as CD14, TNF-alpha, IL6, and MCP-1) and HSCs (such as *α*-SMA and collagen type 1); as a consequence, decreased macrophage and HSC/MFB recruitment has also been shown in damaged areas. So Gas6 deficiency, by limiting cytokine/chemokine release, prevents hepatocyte proliferation, macrophage infiltration in liver necrotic areas (which, in turn, is mediated by a direct chemotactic effect of Gas6), and accumulation of myofibroblasts in healing areas. Interestingly, in Gas6 KO mice, a positive feedback on Axl expression was observed, with the concomitant induction after CCl_4_ treatment of the suppressor of cytokine signaling (SOCS) 1, suggesting that the delayed liver repair in deficient mice may be a consequence of an inhibitory signal arising from Axl receptor overexpression [88].

A similar mechanism probably explains what has been described in hepatic I/R models. As already mentioned, in mice following I/R exposure, an early increase in serum Gas6 levels was reported. Unlike wild-type mice, Gas6-/- mice were highly sensitive to partial hepatic I/R, with 90% of mice dying within 12 hours of reperfusion due to massive hepatocellular injury. I/R induced early hepatic AKT phosphorylation in wild-type but not in Gas6-/- mice, whereas hepatic IL-1*β* and TNF mRNA levels (e.g., lipopolysaccharide- (LPS-) induced cytokines) were higher in Gas6-/- mice compared to wild-type mice. In line with the in vivo data, in vitro studies indicated that Gas6 induced AKT phosphorylation in primary mouse hepatocytes protecting them from hypoxia-induced cell death, while Gas6 diminished IL-1*β* and TNF in murine macrophages. Finally, the protective role of Gas6 on cell growth and survival during tissue repair was confirmed by the fact that in vivo recombinant Gas6 treatment not only rescued Gas6 knockout mice from I/R-induced severe liver damage but also attenuated hepatic damage in wild-type mice following I/R. Thus, it may be speculated that Gas6 could emerge as a potential therapeutic target to reduce postischemic hepatic damage [89].

Synthesizing to the fullest extent, the protective role of Gas6/TAM on the liver is mediated by its strong profibrogenic potential. However, as in all biological processes, even an initially favorable action—like a physiological reparative process—can become, if out of control and especially if protracted in time, a factor of damage itself, since an excessive fibrotic apposition in the liver can in turn become a pathophysiological mechanism of hepatic injury. In this sense, Gas6/TAM has a role like that of a “two-faced Janus,” depending on clinical contexts. These concepts will be further clarified in the following paragraphs.

### 2.3. Role of Gas6/TAM in Liver Fibrosis

The Gas6/TAM system has recently emerged as an important player in progression to liver fibrosis through the aforementioned control of inflammation and liver repair. Not surprisingly, the focus of the few pathophysiological studies currently available is the modulation of HSC activation, because of its recognized role in the liver fibrosis associated to chronic liver injury of any etiology, being HSCs the main collagen-producing cells in any damaged liver [3, 4]. The most convincing study comes from a murine model of Barcena et al. The authors used both a genetic model of Axl deficiency (Axl KO) and a pharmacologic approach, the Axl inhibitor BGB324. HSCs were obtained from wild-type and Axl−/− mice, treated with recombinant Gas6 protein (rGas6), Axl siRNAs, or BGB324, and analyzed by western blot and real-time PCR. Experimental fibrosis was then studied in CCl_4_-treated wild-type and Axl−/− mice and in combination with Axl inhibitor. After five weeks of CCl_4_ treatment, wild-type mice exhibited a marked increase in Gas6 and sAxl serum levels compared to controls, indicating that this pathway is upregulated during CCl_4_-induced liver fibrosis. In primary mouse HSCs, Gas6 and Axl levels paralleled HSC activation. rGas6 phosphorylated Axl and AKT prior to HSC phenotypic changes, while Axl siRNA silencing reduced HSC activation. Moreover, BGB324 blocked Axl/AKT phosphorylation and diminished HSC activation. In addition, Axl KO mice displayed decreased HSC activation in vitro and liver fibrogenesis after chronic damage by CCl_4_ administration. Similarly, BGB324 reduced collagen deposition and CCl_4_-induced liver fibrosis in mice [90].

Based on these premises, it is not hazardous to hypothesize that the Gas6/TAM system may have a prominent role in the pathogenesis of major chronic liver diseases, with particular reference to fibrosis development. However, it must be said that up to now the amount of evidence is still rather scarce, and further clinical studies of adequate potency are needed.

#### 2.3.1. Gas6/TAM System and Nonalcoholic Fatty Liver Disease

Taking into account the aforementioned limitations, one of the most important liver disease models which has been studied is NAFLD, which includes simple nonalcoholic fatty liver and the more serious nonalcoholic steatohepatitis (NASH). This nosological entity is one of the leading causes of liver-related morbidity and mortality, at least in developed Western countries. Whatever its etiology, it is characterized by fat storage in hepatocytes, lobular inflammation, elevated local and systemic cytokines, activation of HSCs, and expansion of LPCs in periportal areas, both in animal and human models [91, 92]. NAFLD is a risk factor associated with toxic and metabolic fatty liver disease and can progress to end-stage cirrhosis [93]. According to the two-hit model of NAFLD, steatosis is the first hit that increases hepatocyte vulnerability to any secondary insult eliciting an inflammatory response, but most probably, both events are tightly interconnected since fat accumulation per se induces oxidative injury and inflammatory cytokine synthesis [94]. The persistent low-grade inflammation due to chronic hepatocyte damage also plays a critical role in LPC expansion, which may play a part in fibrosis [91, 92, 95, 96].

To address the role of Gas6 in NAFLD and in the progression to liver fibrosis, an animal model was studied, e.g., Gas6 KO mice fed with a choline-deficient ethionine-supplemented diet (CDE) or receiving a CCl_4_ treatment [97]. Gas6 deficiency attenuated hepatic steatosis by limiting CDE-induced downregulation of genes involved in *β*-oxidation observed in wild-type animals. Moreover, Gas6-deficient mice displayed a reduction of hepatic inflammation, revealed by limited F4/80-positive macrophage infiltration, decreased expression of IL-1*β*, TNF-*α*, MCP-1, and lymphotoxin-*β*, and attenuated LPC response to CDE diet. Gas6 deficiency moreover reduced CDE-induced fibrogenesis and hepatic myofibroblast activation, decreased expression of TGF-*β* and collagen type 1 mRNAs, and increased Axl protein levels. After chronic CCl_4_ injury, Gas6-deficient mice also exhibited reduced liver fibrosis as a consequence of defective macrophage recruitment compared with wild-type animals. The authors concluded that the improvement of steatohepatitis and fibrosis in Gas6−/− mice was linked to an inhibition of the liver inflammatory response (similar to other previously mentioned models) which in turn regulates lipid metabolism and macrophage recruitment. Thus, this study highlights the possible deleterious effect of Gas6 in the progression of steatosis to steatohepatitis and fibrosis. However, it has to be mentioned that in this CDE model no induction of SOCS1 and 3 could be observed, as previously observed in the CCl_4_ acute model of liver injury [98], thus making the functional relevance of Axl overexpression in Gas6-deficient mice still uncertain. Another possible weakness of the work is that the other components of the TAM family (e.g., Tyro3 and MERTK) were not tested, though they may contribute to the Gas6 effects described in this NAFLD animal model.

While, to the best of our knowledge, no current data are available on Tyro3 role in NAFLD-related fibrogenesis in vivo or in vitro, there are some pieces of evidence about the pathophysiological role of Axl and MERTK.

For what concerns the former one, its distinctive subcellular signaling during NASH development and the efficacy of its intervention to prevent diet-induced liver fibrosis remain to be explained. However, there are several preliminary pieces of evidence that indicate that it may play an important role in NAFLD progression. In particular, in a letter from Mari et al., it is commented that increased Axl levels have been detected in mouse models of NASH, anticipating a significant role for Gas6/Axl in human NASH pathology [98]. These data were recently confirmed in a research from Tutusaus et al. in which it was described how Axl expression was elevated in NAFLD patients and in mouse models of NASH. Among individuals with different degrees of NAFLD (steatosis/fibrosis/cirrhosis), only cirrhotic patients displayed increased Gas6 and MERTK serum levels. However, Axl values were significantly elevated in all NASH groups in parallel to disease progression. Consistent with Axl influence in HSC transdifferentiation, in human activated HSC cells (LX2), the expression of profibrogenic genes after Axl activation was blocked by the selective Axl inhibitor BGB324. Axl control of inflammatory response was then analyzed in activated human THP-1 macrophages. While Gas6 reduced LPS-induced gene expression, Axl inhibition did not affect it. Finally, mice fed with a high-fat diet choline-deficient with methionine restriction (HFCD) developed significant hepatic steatosis and fibrosis and exhibited increased sAxl levels, recapitulating human NASH observations. Besides inhibiting Axl, BGB324 administration increased circulating Gas6, favoring Gas6 liver protection. This protective effect was confirmed also in HFCD-fed mice which showed reduced liver fibrosis and hepatic inflammation. Taken together, these data seem to suggest that sAxl levels are an early marker of NASH that correlates with disease development and, at least in experimental NASH models, that therapeutic inhibition of Axl can diminish liver fibrosis by blocking HSC activation and reducing hepatic inflammation, possibly due to Gas6 hepatoprotective action [99].

The other TAM receptor which has been studied in this disease is MERTK. The latter one is a well-known key component for the initiation of efferocytosis [42, 100] and is overexpressed in mouse HSCs activated by culture in plastic and in experimental models of liver fibrosis (e.g., after induction of chronic liver damage in response to CCl_4_ administration or bile duct ligation) [90, 101]. Moreover, agonists of LXR, a nuclear receptor favoring lipogenesis, increase MERTK expression in monocytes [102]. Therefore, MERTK and its variants could act as central players in the control of apoptosis, immune response, HSC activation, and steatosis modulation, e.g., all factors involved in the pathogenesis of NAFLD and its fibrotic progression to steatohepatitis and cirrhosis. Based on a genome-wide study in patients with CHC which, amongst several susceptibility loci for severity and progression of liver fibrosis, identified as the strongest one the homozygosity for rs4374383 G>A single nucleotide polymorphism, a non-coding variant in the MERTK gene [103], an in vivo and in vitro study was conducted on NAFLD. In a large cohort of patients with histological diagnosis of NAFLD, the protective AA genotype was associated with lower MERTK hepatic expression (fibrosis F2-F4 in 19% of patients with MERTK AA compared to 30% of those with MERTK GG/GA); the AA genotype remained associated with clinically significant fibrosis also at multiple logistic regression analysis. Similar results were observed also when considering severe fibrosis (F3-F4) as histological outcome. The prevalence of NAFLD was not affected by MERTK genotype (39.7% in MERTK AA vs. 44.1% in MERTK GG/GA), but severe steatosis was observed in 8% of patients with MERTK AA compared with 21% with MERTK GG/GA genotype. Again, MERTK AA genotype remained associated with severe steatosis at multiple logistic regression. MERTK was overexpressed in the liver of NAFLD patients with F2-F4 fibrosis, mainly in HSCs and macrophages (but not in hepatocytes). Similarly, the receptor was more represented in an animal model of fibrogenesis (e.g., mice fed with a methionine- and choline-deficient diet). Furthermore, the exposure of cultured human HSCs to the MERTK ligand Gas6 increased cell activation and migration and induced the expression of the profibrogenic procollagen I. These effects were counteracted by the inhibition (both with specific small molecule inhibitor UNC569 and siRNA) of MERTK activity, which also resulted in apoptotic death of HSCs. The results of this research seem to provide sufficient evidence for considering MERTK AA genotype as an appealing new genetic biomarker in natural history, pathophysiological, and interventional studies in NAFLD [98, 104, 105].

#### 2.3.2. Gas6/TAM System and Other Liver Diseases

Taking into account other liver disease models, some preliminary in vivo data are available for ALD and CHC infection. In the previously mentioned Barcena et al. paper, the authors recruited a small sample of patients (30 ALD, 51 CHC) who all had hepatic fibrosis staged by liver biopsy. Additionally, in both groups, patients were evenly distributed in regard to the different degrees of liver disease, although in the ALD group no moderate fibrosis cases (METAVIR F2-F3) were included. Gas6 and sAxl serum levels were measured before initiation of treatments. In ALD patients, both an increase of Gas6 and sAxl were found in serum levels of cirrhotic patients, showing close correlation to the severity of the disease, although behaving differently. Specifically, sAxl concentration had already augmented in individuals with compensated cirrhosis compared to initial fibrosis, while Gas6 levels had increased markedly in the decompensated cirrhosis group. Moreover, a remarkable correlation was found between the MELD score and both proteins. In CHC patients, Gas6 levels were significantly different among individuals with established fibrosis (F2) and patients with initial fibrosis (F0 and F1 groups). In addition, F2 fibrosis patients' sAxl levels displayed significant changes in comparison to individuals with advanced fibrosis or cirrhosis (F3/F4 group). The authors therefore could provide groundbreaking evidence emphasizing for the first time the relevance of the Gas6/Axl pathway also during the development of ALD- and CHC-induced liver damage, supporting Gas6 and sAxl serum levels as indicative parameters of hepatic dysfunction and fibrosis development in liver disease and suggesting their possible future prognostic role within a patient multidimensional evaluation [90].

The report that sAxl levels are increased in advanced fibrosis/cirrhosis has been confirmed in a much wider population including 75 healthy controls, 400 chronic liver disease patients of various etiologies (chronic viral hepatitis, autoimmune hepatitis, cholestatic liver disease, and NAFLD) and fibrosis stages (including cirrhosis), and 347 HCC patients [106]. For cirrhosis, sAxl showed a sensitivity of 80.8% and a specificity of 92.0% at a cutoff of 54.0 ng/ml.

In any case, Axl is not the only component of the TAM family which has demonstrated a putative role in hepatic fibrosis progression. For instance, there are growing pieces of evidence of MERTK involvement, at least for what concerns CHC. More in detail, a genetic predisposition with regard to an accelerated fibrosis (demonstrated by liver histology and/or transient elastography) has been reported for what concerns the aforementioned rs4374383 G>A single nucleotide polymorphism. As shown in other diseases, it is likely that patients carrying the GG/GA genotypes have a significantly higher hepatic MERTK expression, although the underlying mechanism is unknown [104]. This in turn will lead to a dysregulation of the phagocytosis of apoptotic cells by macrophages and, more in general, of various inflammatory responses [103, 107–109]. It has to be noted that, although the rs4374383 SNP is not located in a regulatory MERTK region, a high number of SNPs are in high linkage disequilibrium (LD) with it. Thus, another SNP or SNPs in high LD could be causally responsible. This issue was investigated by Cavalli et al., who suggested that rs6726639A allele, in high LD with rs4374383 (*r*^2^ = 0.94), could promote the binding of interferon regulatory factor 1 (IRF1) to this region [110] and serve to activate or repress the expression of a high number of genes involved in the immune response [111]. The preferential binding of IRF1 to the A allele compared to the C allele would downregulate MERTK in patients carrying the A allele, protecting against CHC liver fibrosis and HCC. So, in genetic association studies, the two SNPs (rs4374383 and rs6726639) may be interchangeable for predicting liver fibrosis progression.

The results of the aforementioned studies could leave room to a possible future role for the targeting of TAM receptors (e.g., with small molecule inhibitors against Axl or MERTK) as a therapeutic strategy for liver fibrosis management, with the caveat that any such therapeutic approach might face toxicity. The measurement of the soluble levels of Gas6 and its receptors (e.g., sAxl and sMERTK [102]) could furthermore be the basis of providing an easy and accurate measurement of hepatic fibrosis progression, since numerous other targets for antifibrotic agents are difficult to be analyzed or to enter early-phase clinical studies due to the lack of sensitive markers to follow the effects [90].

Returning to clinical studies whose purpose is to test the role of plasma Gas6 as a novel putative biomarker of hepatic fibrosis in different disease models, a paper from Bellan et al. deserves a mention [112]. A fair number (113) of patients were studied, the vast majority (81%) being affected by CHC infection. Fibrosis was staged by transient elastography and/or, whenever feasible and accepted, by liver biopsy; again, all stages of hepatic disease were represented, from initial fibrosis to decompensated cirrhosis. Authors confirmed Barcena's finding that patients with histological demonstration of severe fibrosis had significantly higher Gas6 plasma concentrations; they were also able to demonstrate for the first time that Gas6 plasma concentration was directly correlated to liver stiffness measured by transient elastography. Even more relevant, the diagnostic accuracy of Gas6 was comparable to that of liver elastography both in ruling out and in detecting severe liver fibrosis. A proposed threshold of 30 ng/ml for Gas6 plasma concentration was able to rule out a clinically relevant degree of fibrosis with an 84% sensitivity and 56% specificity, while values >42 ng/ml identified severe fibrosis with a sensitivity of 64% and a specificity of 95%; however, taking into account that the majority of patients was affected by chronic viral hepatitis, some caution should be exercised before automatically generalizing these conclusions when other conditions can be factors.

#### 2.3.3. Gas6/TAM System: Does It Have a Role in Fibrosis Complications?

Of noteworthy importance, in the previously reported paper from Bellan et al. [112], the authors noted a nonstatistically significant trend toward higher Gas6 concentrations in patients affected by cirrhosis complications (e.g., esophageal varices and HCC). These reports are to some extent the expected consequence of the association with the severity of fibrosis, since both conditions complicate the natural history of cirrhosis. The former ones are the direct consequence of a major hepatic fibrosis complication, e.g., portal hypertension.

The linkage with the latter is biologically more complex to explain and also remains plausible for several relevant reasons. The Gas6/Axl (both in transmembrane and soluble forms) system has been, for instance, claimed to be connected to the promotion of tumor invasion in various solid malignancies, as recently confirmed in a meta-analysis conducted on 3,344 total patients (379 with HCC) from 25 studies. Axl overexpression was significantly correlated with poor prognosis (2.03-fold increase in mortality in all solid tumor patients); in a subgroup analysis of different cancer types, Axl overexpression was correlated with shorter overall survival in a few tumors, including HCC (combined HR of 1.89 (95% CI 1.37–2.60, *p* < 0.001)) [113]. The pathophysiological rationale of Gas6/Axl deleterious role probably consists in its capacity to activate HSCs and modulate hepatocyte differentiation, as suggested by a preliminary study which demonstrated that in HCC cancer cell lines Gas6/Axl can enhance cell invasiveness through transcriptional activation of Slug which induces epithelial to mesenchymal transition (EMT) [114]. Notably, under physiological conditions, Gas6 and its receptor Axl are not expressed in hepatocytes. However, Axl is strongly expressed in malignant hepatocytes of about 40% of HCC patients showing progression towards metastasis [115]. Moreover, as previously described in immune stem and cancer cells, EMT-transformed hepatocytes upregulate the expression of Axl and secrete Gas6 revealing a possible autocrine/paracrine regulation loop in the Gas6/Axl pathway [116]. In the background of fibrosis, sinusoidal endothelial cells, activated HSCs, and Axl-positive myofibroblasts as well as Kupffer cells release Gas6 into the tumor microenvironment of HCC, causing a Gas6-enriched HCC stroma. These data suggest that Axl signaling drives HCC progression in the presence of large amounts of bioactive Gas6 and is of even more particular interest as tyrosine kinase inhibition is one of the most exploited antitumoral approaches of targeted therapies (e.g., bosutinib) [114, 117, 118]. However, since very complex mechanisms are involved that go extensively beyond the simple induction of hepatic fibrosis, the precise analysis of the possible oncogenic roles of the Gas6/TAM system in HCC signaling (in many cases still lacking solid evidence) remains outside the purpose of the present review.

A further analysis of the possible association of Gas6 plasma concentrations with the presence of esophageal varices comes from the same research group which extended the abovementioned preliminary finding in a large cohort of CHC-infected cirrhotic patients [119]. The clinical rationale for such a research is that early detection of patients with varices at high risk of bleeding (e.g., large varices) is crucial in cirrhotic patients, but sparing endoscopy to low-risk patients would be worthy of consideration. With this in mind, noninvasive methods, such as Baveno VI criteria, have been proposed to stratify the risk of esophageal varices (suggested cutoffs for which screening for esophageal varices can be safely omitted: liver stiffness at transient elastography < 20 kPa and a platelet count > 150 × 10^9^/l) but unfortunately have some limitations [120]. In the studied cohort, a total of 74/160 (46%) patients had esophageal varices that were large in 17/160 (11%) cases. 34/160 patients (21%) satisfied Baveno VI criteria to avoid variceal screening, but one of them had large varices at upper gastrointestinal endoscopy (sensitivity 94%). Serum Gas6 values increased from 63 ng/ml among patients without varices to 75 ng/ml among patients with small varices and to 98 ng/ml among those with large varices. A plasma Gas6 value < 45 ng/ml, detected in 34/160 (21%) patients, was 94% sensitive (but only 23% specific) in identifying patients without large varices; one of these patients (different from the subject missed by Baveno VI criteria) had large varices at upper gastrointestinal endoscopy. The authors could then conclude that plasma Gas6 concentration is a highly sensitive test to identify patients with large varices, outperforming the platelet count as a single biomarker of large varices and proving to be comparable to the diagnostic performance of Baveno VI criteria. This could provide the initial rationale for a future role for Gas6 in clinical settings in which liver elastography is still not available or in those patients for whom a reliable liver stiffness cannot be obtained (e.g., for ascites or morbid obesity).

For what concerns other severe complications of cirrhosis, e.g., portal hypertension-induced ascites, the role of other TAM system members has been demonstrated, with particular regard to MERTK. This prorestorative marker shows a two-faced activity: while for instance it is abundantly expressed in liver macrophages during the resolution phases of several diseases (e.g., acetaminophen-induced liver injury) [121], it has also been identified as a potent suppressor of T cell responses [122]. Regarding the latter activity, there are for instance some pieces of evidence about the development of immunoparesis in patients with acute on-chronic liver failure (ACLF) involving the unbalanced activation and overexpression of MERTK on monocytes/macrophages in the circulation and tissue sites of inflammation [123–125]. The great influence of MERTK-positive monocytes was confirmed in a late Antoniades work that studied ACLF patients with ascites. Immunometabolic profiling of their ascites revealed profound disturbances in myeloid cells with upregulated MERTK expression, impaired proinflammatory responses to LPS, preferential lipid metabolism, and evidence of epithelial cell death. The impact of these perturbations on bacterial clearance could predispose to an increased susceptibility to infections such as spontaneous bacterial peritonitis (another severe complication of cirrhosis), but this still requires further exploration [126].

Notably, coming back to HCC, some preliminary data exist on a similar pattern of severe myeloid impairment. As a matter of fact, in this tumor, it has been reported the expansion of a MERTK-expressing immunosuppressive tumor-associated macrophage population that suppresses host innate and adaptive immune responses. In the same study, neoplastic patients, compared with controls, had also a significant increase in MERTK-expressing circulating monocytes (and in Gas6, as previously mentioned). Inhibition of MERTK signaling restored their proinflammatory capabilities, thereby identifying a possible novel immunotherapeutic target in HCC [127].

#### 2.3.4. Possible Implications of Soluble Axl in Liver Fibrosis

As previously mentioned, Axl can be cleaved and released in serum as sAxl. Since the latter is still able to bind Gas6 and is therefore capable of depleting the ligand, it is considered to be a critical determinant in affecting autocrine or paracrine Axl signaling [128, 129]. Consequently, hepatic fibrosis progression should be subsequently attenuated by diminished Gas6/Axl signaling, resulting in a phenotype comparable to the one of chemically challenged Gas6 KO mice [97]. However, serum Gas6 levels have been shown to be elevated in patients with advanced fibrosis and cirrhosis as well as in HCC patients [85, 101]. Moreover, high Axl expression as well as high sAxl levels independently correlate with fibrosis/cirrhosis [90, 106, 130]. These findings are contradictory to the hypothesis that ectodomain shedding of Axl only leads to signal dampening. There is in fact evidence that the ICD of Axl could remain active supporting the belief of both a Gas6-independent signaling, in parallel with a Gas6-dependent one, which has been revealed by the previously mentioned Gas6 KO and Axl KO studies showing reduced fibrogenesis [90, 97]. This hypothesis is supported by the Holstein et al. group that proposed there might be a switch predisposing liver fibrosis, cirrhosis, or even HCC development, where even in the event of a cleavage of Axl, the inhibitory Axl shedding mechanism is circumvented due to the presence of abundant nonshedded Axl receptors that will overcome the loss of proteolytically cleaved Axl. Available free Gas6 is then able to bind increasingly expressed Axl receptor and stimulate Gas6/Axl signaling driving fibrosis in the liver [117]. This Gas6-independent signaling hypothesis implicates that proteases are recruited to cleave the Axl ectodomain after Gas6-mediated Axl activation. In this scenario, the ICD could remain active and could still be able to phosphorylate effector molecules [117]. However, it is an open question as to whether ectodomain shedding occurs after Axl homodimerization and ICD activation. Interestingly, a mechanism of shedding prior to receptor activation with ligand-independent signaling has been reported for ErbB2 [131].

## 3. Conclusions

In the present paper, we have reviewed current evidence regarding the use of Gas6 and its TAM receptors as potential biomarkers of liver fibrosis.

The rationale for interest in Gas6 system derives from the proven role of the Gas6 pathway in the HSC transdifferentiation process from a normal vitamin A-storing to an ECM-remodeling phenotype. This indeed is what initializes fibrosis. Despite recent progress in understanding the biology of HSCs, the mechanisms are not yet fully understood. In fact, in addition to the treatment/withdrawal of the underlying cause, fibrosis regression in chronic liver diseases is not accomplished by any antifibrotic drug despite the experimental description of an array of pharmacological targets [3, 6]. Based on these premises, the exact biological roles of Gas6 pathways, though undoubtedly relevant in human liver pathology, are still under investigation at least in regard to the fibrogenesis process. At any rate, what has clearly emerged from preclinical and clinical studies is that Gas6/TAM is a profibrogenic route. This means that it is beneficial/reparative in the event of acute damage but profibrogenic/harmful if the insult chronicizes. In this context, not only does the evidence available so far make it interesting to test the potential use of these system-related proteins as serological markers of disease progression/fibrosis but we may also speculate that this pathway may provide a new therapeutic target not only for liver fibrosis but also for different chronic liver diseases. Moreover, the existence of specific inhibitors [132, 133], already in clinical trials, may facilitate the biomedical translation of preclinical studies.

In the current state of the art, in essence, a sufficient amount of data has now accumulated showing that Gas6 and its receptors, such as Axl and MERTK, play a relevant role in the major models of chronic liver diseases. However, reference has been limited to approximately a few hundred patients tested in vivo. While results of the studies conducted to date are promising, some major drawbacks remain. First, to prove accurate staging of liver fibrosis by these novel serological markers, liver biopsies will still be needed to identify the specific stage of fibrosis in each patient. The lack of such material results in the usual incapacity of most studies to report other facets of biomarkers beyond the ability to identify late stage fibrosis or cirrhosis, as compared to transient elastography or validated serological scoring algorithms. Second, large-scale multietiology validation of such novel serum markers is still needed. The efficiency of the biomarkers should be tested prospectively on large patient cohorts with differences in age, gender, etiology of liver disease, etc. Moreover, it is reasonable that these novel biomarkers might find, as many other noninvasive analytes, their best use within more complex algorithms rather than in the simple measurement of their plasma concentrations. For example, in the paper from Barcena et al., an algorithm containing sAxl and Gas6 could achieve an even stronger correlation (*r*^2^ = 0.86) with the MELD score than the two analytes taken individually, suggesting that the measurement of both proteins provides a better evaluation of liver functionality [90]. Most likely, however, the best diagnostic solution will be achieved combining these markers with more variables, not necessarily directly related to the fibrosis itself. A first preliminary confirmation comes from the work of Staufer et al. in which sAxl performed better in predicting advanced liver fibrosis (≥F2) when combined with serum albumin (in a sAxl/albumin ratio) in various chronic liver diseases [130].

Finally, the fine mechanisms of this like pleiotropic system have still to be fully clarified. First of all, an important issue about published data is that most have involved the assay of single ligand against single TAM receptor. However, some analysis has demonstrated that invalidation of one TAM receptor might induce a compensatory enhancement of one or two other TAM receptors and also vice versa in the case of upregulation of a single receptor [134, 135]. However, the functional consequences of this reciprocal regulation remain unclear. The complexity of Gas6/TAM system is also revealed by what happens when it is the ligand (e.g., Gas6) to be deficient. In these cases, a high and constitutive expression of Axl is found, which reveals a negative control exerted by Gas6 on its high-affinity Axl receptor expression. However, the functional relevance of Axl overexpression, at least in Gas6-deficient mice, is still uncertain and deserves further studies [88, 97]. Another Axl negative feedback regulation, which needs to be better clarified in liver pathology, involves microRNA (miRNA). There are some preliminary data in tumoral cells showing that miR-34a may target the 3′ UTR of Axl mRNA to posttranscriptionally inhibit Axl expression, modulating apoptosis in cancer cells, and revealing functional implication of miRNA in the carcinogenic process. On its turn, Axl overexpression may induce miR-34a expression [136]. Obviously, also many positive feedbacks regulate Gas6/TAM system. There is for instance an interesting recent report that again needs to be validated in hepatic diseases about a novel oncogenic long noncoding antisense RNA (Gas6-AS1) that can control the expression of its cognate sense gene Gas6 at the transcriptional or translational levels. Its net effects consist in supporting tumor progression via inducing an increase of Axl levels and driveling Axl signaling pathway activation [137]. Another partially unresolved issue concerning Gas6/TAMs is, as previously mentioned, whether the shedding of activated Axl receptors could lead to Gas6-independent signaling, with potential consequences not only on fibrogenesis but also on hepatic oncogenesis [117]. Other issues which need further investigation concern the other relevant TAM receptor in liver pathology, e.g., MERTK. As a matter of fact, it still has to be resolved the particular contribution of sMERTK to hepatic inflammation and fibrogenesis. It is believed, like other soluble TAM receptors, to compete with the bound receptors and thus inhibit their function. In other chronic disease models, significantly lower expression of MERTK in monocytes has been described; conversely, sMERTK expression was increased compared to controls and related to disease severity. Moreover, the metalloproteinase ADAM 17, responsible for cleavage of MERTK to its soluble form, has been shown to be increased in patient monocytes [138]. These observations suggest that functional deficiency of TAM receptor-mediated regulation of inflammation may contribute to chronic inflammation and, translating this to liver physiopathology, be a potential driver of fibrosis progression. It would then be interesting to evaluate if sMERTK levels are altered in patients with steatohepatitis or viral infection. However, additional aspects of MERTK liver biology deserve, in our opinion, to be further analyzed. For example, future research should verify if MERTK inhibition or MERTK KO mice display reduced fibrosis, as already observed in Axl KO mice or after pharmacological Axl inhibition [90].

In conclusion, in the foreseeable future, Gas6/TAM receptors have a strong pathophysiological rationale and a potential use as serological markers of disease progression in chronic liver diseases; moreover, the system may be targeted in future liver therapies (e.g., in clinical trials testing small molecule inhibitors). If these tools were to be further optimized by improving their accuracy, while at the same time handling other possible confounding factors, their presence in a liver clinic may provide a means for making the correct diagnosis, analogous to having a much longed for crystal ball.
